# P-1712. Aspergillus Osteomyelitis: Clinical Features, Management, and Outcomes Across Two Large U.S. Centers

**DOI:** 10.1093/ofid/ofaf695.1884

**Published:** 2026-01-11

**Authors:** Adam G Stewart, Carlos A Portales Castillo, Miriam Barshak, Sarah P Hammond

**Affiliations:** University of Texas MD Anderson Cancer Center, Brisbane, Queensland, Australia; Massachusetts General Hospital, Boston, Massachusetts; Harvard Medical School, Massachusetts General Hospital, Boston, MA; Massachusetts General Hospital, Boston, Massachusetts

## Abstract

**Background:**

*Aspergillus* osteomyelitis is a rare form of invasive aspergillosis (IA), and its epidemiology, clinical manifestations, radiographic features, management, and outcomes remain poorly defined.

**Figure:**
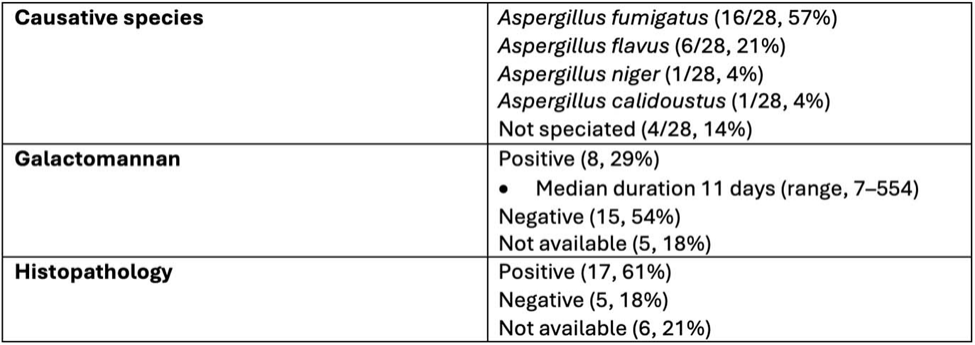
Microbiological characteristics

**Methods:**

Utilizing the Mass General Brigham’s Research Patient Data Registry, a query for cases of *Aspergillus* osteomyelitis at both Massachusetts General Hospital and Brigham and Women’s Hospital from January 2000 to January 2023 was performed. Individual patient charts were reviewed for demographic and clinical data, microbiology, radiology, as well as surgical management, antifungal therapy and outcomes. Included patients were adults ( >18 years) with either proven or probable IA, as defined by EORTC/MSGERC, involving bone.

**Results:**

Twenty-eight patients were included. Median age was 68 years (range 20-84 years) and 71% were male. Predisposing conditions included thoracic solid organ transplant (SOT) (25%), hematologic malignancies (21%), diabetes mellitus (18%), solid malignancies (11%), concurrent head and neck infections (11%), immunosuppression not otherwise specified (7%), trauma or prior surgical intervention (7%). The most common sites of infection were sternum (32%), skull base (18%), lower extremities (18%) and vertebrae (14%). All patients with sternal osteomyelitis (n=9) had undergone thoracic surgery within 12 months prior to diagnosis. The vast majority (86%) of patients had positive cultures for *Aspergillus* species. The most common species identified was *A. fumigatus* (54%) followed by *A. flavus* (21%). Imaging (CT or MRI) was compatible with the diagnosis of osteomyelitis in 64% (n=20) cases. Serum galactomannan was checked in 82% (n=23) cases and found to be positive in 8 (29%). In two cases, serum galactomannan remained positive for more than 120 days. Combined surgical and antifungal management was performed in most cases (79%). Voriconazole was the most common antimicrobial used for definitive therapy (68%). 1-year all-cause mortality was 29%.

**Conclusion:**

Important clinical outcomes, such as mortality, may be more favorable when compared to other forms of acute IA and could relate to localized disease which can be surgically resected, or host factors. Immunosuppressive conditions are predictably overrepresented in patients with IA involving bone.

**Disclosures:**

All Authors: No reported disclosures

